# Iron Brain Menace: The Involvement of Ferroptosis in Parkinson Disease

**DOI:** 10.3390/cells11233829

**Published:** 2022-11-29

**Authors:** Kai-Jung Lin, Shang-Der Chen, Kai-Lieh Lin, Chia-Wei Liou, Min-Yu Lan, Yao-Chung Chuang, Pei-Wen Wang, Jong-Jer Lee, Feng-Sheng Wang, Hung-Yu Lin, Tsu-Kung Lin

**Affiliations:** 1 Center for Mitochondrial Research and Medicine, Kaohsiung Chang Gung Memorial Hospital, Kaohsiung 83301, Taiwan; 2 Department of Family Medicine, National Taiwan University Hospital, Taipei 100225, Taiwan; 3 Department of Neurology, Kaohsiung Chang Gung Memorial Hospital, Chang Gung University College of Medicine, Kaohsiung 83301, Taiwan; 4 Center of Parkinson’s Disease, Kaohsiung Chang Gung Memorial Hospital, Kaohsiung 83301, Taiwan; 5 Department of Anesthesiology, Kaohsiung Chang Gung Memorial Hospital, Chang Gung University College of Medicine, Kaohsiung 83301, Taiwan; 6 Department of Neurology, Pao Chien Hospital, Pingtung 90064, Taiwan; 7 Department of Biological Science, National Sun Yat-Sen University, Kaohsiung 80424, Taiwan; 8 Department of Metabolism, Kaohsiung Chang Gung Memorial Hospital, Chang Gung University College of Medicine, Kaohsiung 83301, Taiwan; 9 Department of Ophthalmology, Kaohsiung Chang Gung Memorial Hospital, Chang Gung University College of Medicine, Kaohsiung 83301, Taiwan; 10 Department of Medical Research, Kaohsiung Chang Gung Memorial Hospital, Kaohsiung 833, Taiwan; 11 Research Assistant Center, Show Chwan Memorial Hospital, Changhua 500, Taiwan

**Keywords:** Parkinson disease, ferroptosis, iron metabolism, lipid peroxidation, system x_c_^−^, glutathione, GPX4, CoQ10, FSP1, lipoxygenase

## Abstract

Parkinson disease (PD) is the second-most common neurodegenerative disease. The characteristic pathology of progressive dopaminergic neuronal loss in people with PD is associated with iron accumulation and is suggested to be driven in part by the novel cell death pathway, ferroptosis. A unique modality of cell death, ferroptosis is mediated by iron-dependent phospholipid peroxidation. The mechanisms of ferroptosis inhibitors enhance antioxidative capacity to counter the oxidative stress from lipid peroxidation, such as through the system x_c_^−^/glutathione (GSH)/glutathione peroxidase 4 (GPX4) axis and the coenzyme Q10 (CoQ10)/FSP1 pathway. Another means to reduce ferroptosis is with iron chelators. To date, there is no disease-modifying therapy to cure or slow PD progression, and a recent topic of research seeks to intervene with the development of PD via regulation of ferroptosis. In this review, we provide a discussion of different cell death pathways, the molecular mechanisms of ferroptosis, the role of ferroptosis in blood–brain barrier damage, updates on PD studies in ferroptosis, and the latest progress of pharmacological agents targeting ferroptosis for the intervention of PD in clinical trials.

## 1. Introduction

Parkinson disease (PD) is a progressive clinical syndrome with neuronal loss owing to various pathogeneses [1]. Clinically characterized by slowness of movement (bradykinesia), rigidity, and tremor, PD is the second-most common neurodegenerative disease affecting millions worldwide. The advancement of modern neuroscience has granted patients with PD with effective motor symptomatic treatment in PD, which is among the greatest achievements for neurodegenerative diseases. However, disability and death due to PD are increasing faster than in any other neurological disorder globally, based on the newsroom report by WHO in 2022 [2]. Cardinal motor symptoms in PD are often heralded by a prodromal period, and non-motor manifestations occur during the disease course, including cognitive decline, autonomic dysfunction, olfactory impairment, sleep disorders, and mood disorders, which also can only be treated symptomatically [3]. There is no cure for PD to date, and therapies to delay or modify disease progression are still lacking. Pathologic features that characterize PD are the progressive dopaminergic neuronal loss in the substantia nigra (SN) and associated brain regions, as well as the presence of intraneuronal protein inclusions called Lewy bodies, which are mainly composed of aggregated α-synuclein protein [4]. Braak’s hypothesis suggested that α-synuclein propagation originates from the lower brain stem to limbic and neocortical areas [5,6], whereas some studies have proposed α-synuclein aggregation to stem from the gastrointestinal tract, spreading via the vagus, or to arise from the olfactory bulb and then get transmitted to the lower brain stem in a prion-like manner [7,8]. Aging is the greatest risk for PD, but a complex interplay between genetics, environmental toxins, and some lifestyle habits is also found to affect PD risk and development [9,10]. 

Although the pathological mechanisms underlying PD are not fully elucidated, an important connection between the organelle mitochondria to PD pathogenesis dates back to 1982. when scientists observed parkinsonism developing after drug addict injection of a new “synthetic heroin” containing the by-product 1-methyl-4-phenyl-1,2,3,6-tetrahydropyridine (MPTP), a specific mitochondrial respiratory chain complex I inhibitor [11]. As the major cellular power generator, mitochondria contain electron transport chains (ETC) on their inner mitochondrial membrane dedicated to the production of adenosine 5′-triphosphate (ATP) through oxidative phosphorylation [12]. The possible roles of mitochondria in PD pathogenesis have also been supported by various genetic evidence as many PD risks genes were found to be associated with crucial mitochondrial functions. These include mitochondrial biogenesis (*ATPase type13A2 (ATP13A2)/PARK9*), morphological dynamic (*PRKN/PARK2, phosphatase and tensin homolog (PTEN)-induced putative kinase 1 (PINK1)/PARK6)*, trafficking (*Leucine-rich repeat Kinase 2 (LRRK2)/PARK8*), and elimination of damaged mitochondria (*Daisuke-Junko-1 (DJ-1)/PARK7, F-box only protein 7 (FBXO7)/PARK15, vacuolar protein sorting 35 (VPS35)/PARK17)* [13,14,15,16,17,18,19]. Although highly efficient in energy production, a small percentage of electrons could leak out from the mitochondrial ETC, especially from complexes I and III, and potentially hazardous reactive oxygen species (ROS) are generated as biological by-products [20,21]. Antioxidative mechanisms are imperative to counter these ROS, especially in high-energy-consuming cells, such as neurons. An imbalance between ROS production and antioxidative detoxification leads to oxidative damage [22]. The residual cellular debris caused by oxidative damage will then be removed through autophagy, a cellular process for removal of unnecessary or dysfunctional components [23]. The process of autophagy requires encircling wastes into double-membraned vesicles and their degradation via the lysosome. Some of the PD risk genes, such as *VPS35*, *GBA*, and *TMEM175*, are also connected to the lysosomal system, which is essential for the breakdown and disposal of aggregated α-synuclein [24]. 

With dopaminergic neuronal loss as a key feature in PD pathology, scientists have been investigating the cause of cell death in PD. Regulated cell death has been discussed during the past decades, and these categories were mostly placed on a spectrum between necrotic cell death and apoptotic cell death. Necrotic cell death is an unprogrammed form of cell death causing instantaneous and catastrophic demise, whereas apoptotic cell death is a programmed form of cell death that is tightly controlled by molecular regulators [25]. In recent decades, several pathways of regulated cell death have been named, among which the newly proposed ferroptosis has gained the spotlight (Table 1). Ferroptosis is a term was first anointed in 2012 by Stockwell et al., who found that the system x_c_^−^ inhibitor, erastin, induces a novel form of iron-dependent cell death that has a unique morphology from other cell death types and does not involve the caspase cascades as did apoptotic cell death [26]. This group screened a customized library of 9500 small organic molecules and found ferrostatin-1 as a potent specific inhibitor for this new type of cell death. Based on previous findings that iron and oxidative stress can induce neuronal death, the same group further demonstrated that glutamate-induced cell death could be inhibited by ferrostatin-1 in rat hippocampal neurons [26]. Since then, studies have identified three main features of ferroptosis: iron dependence, increased lipid peroxidation, and an overwhelmed antioxidative system [27,28]. Iron chelators, antioxidative system x_c_^−^, and antioxidants such as coenzyme Q10 (CoQ10) inhibit ferroptosis, while ferroptosis inducers work in the opposite fashion [27,29]. Lately, the connections between PD and ferroptosis were supported by the correlation of iron accumulation with α-synuclein aggregation and the elevation of lipid peroxidation products in the post-mortem SN of patients with PD [30,31,32,33].

In this review, we will first define different types of cell death pathways, describe the steps leading to ferroptosis, introduce connections of ferroptotic factors in the blood–brain barrier and glia, decipher the association between PD and ferroptosis, and demonstrate the potential of anti-ferroptotic mechanisms as treatment strategies for PD.

## 2. Different Types of Cell Death

Pathological PD features are progressive dopaminergic neuronal loss and Lewy body formation. A constant and relentless downhill trajectory of clinical symptoms troubles patients with PD; however, disease-modifying remedies have still not been found. Understanding how these neurons die and how to prevent the occurrence of cell death is therefore crucial for the development of strategies for curbing PD progression. Cells are considered to die either from an accidental cell death (ACD) or a regulated cell death (RCD) [45]. ACD occurs when cells encounter dramatic environmental or metabolic scenarios, such as stroke or trauma. RCD, on the contrary, is under tight molecular regulation to target the elimination of superfluous, irreversibly damaged, and potentially harmful cells. Although RCD is under strict genetically encoded mechanisms to prevent accidental removal of normal functional cells, uncontrolled RCD may lead to early development of degeneration. Thus, pharmacological or genetic intervention of the molecular pathways involved in RCD may provide space for disease modulation for neurodegenerative diseases, such as PD [46]. Traditionally, necrosis represents ACD as a premature, chaotic, and unorganized demise that occurs from an overwhelming unexpected extracellular noxious stimulus. The characteristic morphology of necrotic cells portrays cell organelles swelling, plasma membrane rupture, cell lysis, and final uncontrolled spillage of inflammatory intracellular contents, causing damage to the surrounding tissue. In contrast, apoptosis is a planned and non-immunologic (tolerogenic) RCD. Renovating this view, in 2008, Hitomi et al. reported that programmed necrosis does exist through mediating a complex molecular pathway [47]. As cell death research progressed, new cell death pathways surfaced with their own molecular initiation and propagation. A considerable degree of interconnectivity was found between these different cell death pathways. According to the Nomenclature Committee on Cell Death (NCCD) 2018, several types of RCDs have been described, including apoptosis, autophagy, necroptosis, pyroptosis, and ferroptosis [25]. The morphological features of these RCDs were placed on a spectrum from fully necrotic to fully apoptotic and also had their immunomodulatory profile ranging from pro-inflammatory and immunogenic to anti-inflammatory and tolerogenic (Table 1) [25]. A further explanation of RCD involves two diametrical scenarios. The first, also known as programmed cell death (PCD), works as a built-in physiological program that combines prewritten genetic information with intracellular or extracellular stimuli to determine the cell fate for its role in the body [48]. These PCDs include development at embryonic stages, tissue turnover, targeted elimination of cells at the risk of neoplastic transformation, or those hijacked by microbes for pathogen replication [49]. Strictly physiological, PCDs do not relate to homeostasis and therefore do not occur in the situation of failing adaptation to stress [25]. The second scenario of RCD occurs in response to prolonged and unrecoverable perturbations of the cellular microenvironment that cannot be stabilized by other means of adaptative cellular homeostasis in order to contain damage to a minimal with limited cell death.

Apoptosis is the first-found and most well-known RCD, meaning “to fall off” in Latin. During the process, cells commit suicide to enable normal development for an individual. There are two major types of apoptosis pathways: the intrinsic pathway (mitochondrial related), activated in response to cellular stresses, such as DNA damage or growth factor deprivation [50], and the extrinsic pathway, which is mediated by death receptors receiving pro-death signals from outside the cell, often by natural killer lymphocytes or CD8-positive cytotoxic T lymphocytes [51]. Both apoptotic pathways are caspase dependent, with activated caspases 3/7 as final executioners, which further activate downstream proteases and nucleases. Because apoptotic cells send “find me” and “eat me” signals to phagocytes, they are typically recognized and engulfed by macrophages before leakage of their intracellular contents. Therefore, apart from limiting direct cell damage caused by the release of cytoplasmic contents, apoptosis typically precludes the release of immune-stimulatory molecules and thus prevents unwanted immune responses [52]. Characteristic apoptotic morphological features include cell shrinkage, cell fragmentation into membrane-bound apoptotic bodies, an intact cell membrane, rapid ingestion by neighboring phagocytic cells, internucleosomal chromatin condensation, and DNA fragmentation by selectively activated DNases [53]. 

A somewhat controversial type of RCD is the autophagic cell death, which is mechanistically dependent on the autophagic machinery, as defined by the 2018 NCCD. Autophagy is the process of cytoplasmic material segregation, its deliverance, and then elimination in the lysosome [54]. This recycling of essential cellular components and degradation of damaged organelles can promote cell survival following stress or nutrient limitation [55]. However, in the excess of autophagy, this biological process is in complex interplays with different forms of cell death. Nicely reviewed by Kumar et al., the roles of autophagy in cell death can be defined as (i) autophagy-associated cell death, where autophagy accompanies cell death induction but does not play an active role in it; (ii) autophagy-mediated cell death, where autophagy induction initiates apoptosis; and (iii) autophagy-dependent cell death, a distinct form of cell death that occurs independently of apoptosis or necrosis [38]. Both genetic and chemical inhibition of autophagy prevents autophagy-mediated cell death by terminology criteria. Additionally, the involvement of at least two different proteins of the autophagy machinery (involved in initiation, nucleation, and elongation) is recommended to establish autophagy-mediated cell death [56]. In the case of autophagy-dependent cell death, stricter rules are applied—not only does autophagy inhibition prevent cell death but also neither apoptosis nor necrosis is involved or proceeds in parallel. Morphological observations of autophagy-dependent cell death show the formation of large-scale autophagic vacuolization, which hold cytosolic materials and organelles in the dying cells [57]. 

Necroptosis is referred to as programmed necrosis and occurs in response to tumor necrosis factor (TNF), Fas, or TRAIL, as well as certain Toll-like receptor ligands, under conditions when the caspase activity for apoptosis is blocked [40]. Since most of the necroptosis triggers are highly pro-inflammatory, damage-associated molecular patterns (DAMPs), cytokines, and chemokines are released during necroptosis, which causes consequential inflammatory responses. In contrast to the robust inflammatory response induced by necrosis, shifting into necroptosis terminates pro-inflammatory responses earlier [58]. Key modulators of the necroptosis signaling pathway are receptor-interacting protein kinase 3 (RIPK3) and its substrate, mixed lineage kinase domain-like protein (MLKL) [39]. The necroptotic morphology displays clustered dying cells, disrupted membranes, swollen cell bodies and organelles (on the necrosis side), fragmented chromatin (on the apoptosis side), and a large quantity of inflammasomes [41].

Pyroptosis, a novel RCD, is defined by the activation of inflammatory caspases and is associated with innate immunity and cancer [59]. The nomenclature of pyroptosis originated in Greek, with “pyro” meaning “fire/fever” and “ptosis” meaning “falling” to describe this pro-inflammatory programmed cell death. The earliest research reporting pyroptosis dates back to 1986 by Friedlander [60]. Induction of pyroptosis requires at least one member of the inflammatory caspases, including caspase-1, caspase-4, and caspase-5 in humans; subsequent inflammasome activation; secretion of cytokines (e.g., IL-1b and IL-18); and gasdermin translocation to the plasma membrane to form membrane pores [61]. Through the ruptured plasma membrane, cellular contents, including DAMPs and cytokines, are released to mount a robust inflammatory response [62]. Meanwhile, intracellular pathogens (bacteria and viruses) are also released from these cells to make these pathogens more susceptible to neutrophils [42]. A characteristic pyroptosis morphology has the apoptotic characteristics of DNA damage, nuclear condensation, and necrotic cell swelling and rapid destabilization of plasma membrane integrity [63].

Lately, ferroptosis, an iron-dependent type of RCD is gaining attraction among researchers. As described by Stockwell et al., and later research, there are three hallmarks of ferroptosis: the loss of lipid peroxide repair capacity by the phospholipid hydroperoxide glutathione peroxidase-4 (GPX4), the availability of redox-active iron, and the oxidation of polyunsaturated fatty acid (PUFA)-containing phospholipids, among which the latter is the main driver of ferroptotic death [26,64]. Since oxidative damage, lipid peroxidation, and iron accumulation in the SN are concomitantly noted in patients with PD, as well as dopaminergic neuronal loss being the main characteristic pathological feature, ferroptosis is rationally considered to be critically involved in the pathophysiology of PD development [65,66]. Details regarding this form of RCD will be elucidated in the following section.

## 3. Ferroptosis: Iron- and Lipid-Peroxidation-Dependent Cell Death

**Figure 1:**
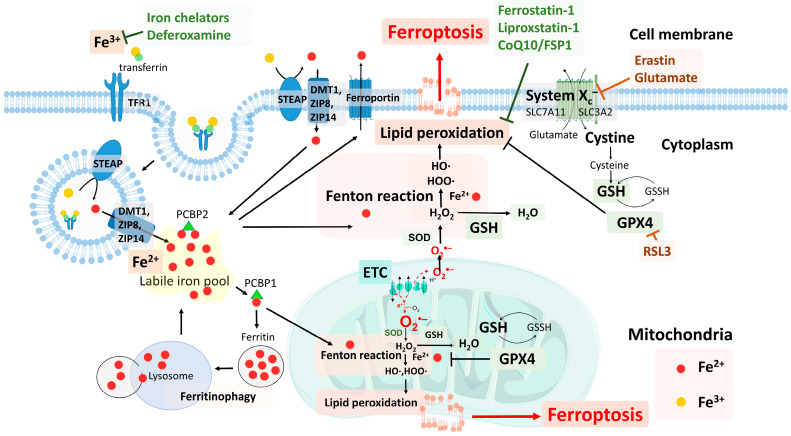
Regulatory pathways of ferroptosis. The iron metabolism (left) includes the endosomal uptake of ferritin-bound Fe^3+^, Fe^3+^ conversion to Fe^2+^ via STEAP proteins, Fe^2+^ release through the transporters DMT1 and ZIP8/14, and Fe^2+^ distribution to different intracellular regions via the chaperones, including PCBP1 and PCBP2. Iron is delivered to the labile iron pool, imported into the mitochondria, stored in ferritin, etc. In low-iron situations, the degradation of ferritin through ferritinophagy occurs and releases the ferritin-bound iron into the cytoplasm. In the case of excess iron, the iron is exported by ferroportin. The mitochondrial ETC generate O2—which is reduced to H_2_O_2_ by the antioxidant SOD. The H_2_O_2_ under the catalysis of Fe^2+^ from the labile iron pool participates in the Fenton reaction, creating hydroxyl radicals, which further activates lipid peroxidation, leading to ferroptosis. The system x_c_^−^/GSH/GPX4 axis (right) exerts antioxidative effects to prevent ferroptosis. Cystine is transported into the cell by system x_c_^−^ and then converted to cysteine, which is a key component of GSH. The reducing activity of GSH keeps GPX4 in its reduced state to maintain the antioxidative abilities of GPX4. GPX4 can directly inhibit lipid peroxidation, therefore inhibiting ferroptosis. Inducers of ferroptosis inhibit system x_c_^−^, such as erastin and glutamate, or inhibit GPX4, such as RSL3. Inhibitors of ferroptosis include iron chelators, such as deferoxamine, and lipid peroxidation inhibitors, such as CoQ10, ferrostatin-1, and liproxstatin-1.

Ferroptosis is an iron-dependent form of RCD driven by enhanced lipid peroxidation and insufficient capacity of thiol-dependent antioxidative mechanisms [27]. Iron is a critical inducer of ferroptosis, without which the cell death pathway may be inhibited, whereas an exogenous supply of iron enhances ferroptosis [67,68]. Unrestrained lipid peroxidation of mainly the PUFA in the membrane bilayers is one of the hallmarks for ferroptosis [69]. The accumulation of lipid peroxides in ferroptosis is iron dependent and can be pharmacologically inhibited by iron chelators (e.g., deferiprone, deferoxamine) and small lipophilic antioxidants (e.g., ferrostatin, liproxstatin, vitamin E, and butylated hydroxytoluene) [7]. In 2003, erastin, an inhibitor of system x_c_^−^, was found to induce non-apoptotic cell death. In 2012, Dr. Brent R. Stockwell first described ferroptosis as a form of regulated iron-dependent cell death that had resulted from the overwhelming accumulation of lethal lipid peroxides in response to erastin/Ras-selective lethal small molecule 3 (RSL-3) administration in a rat brain slice model [26]. The exact mechanisms underlying the execution of ferroptosis have not yet been elucidated. Various inhibitors of apoptosis, necrosis, and autophagy failed to rescue this RSL-induced cell death, but antioxidants and iron chelators, in contrast, prevailed [26]. GPX4 is the primary cellular enzyme that specifically detoxifies phospholipid hydroperoxides to lipid alcohols using glutathione (GSH) as a cofactor [70]. As the rate-limiting amino acid used in the synthesis of the tri-peptide GSH, cystine is essential in the cellular antioxidative defense [71]. The cystine/glutamate antiporter, system x_c_^−^, imports cystine across the cell membrane. Inhibition of cystine uptake by erastin, an inhibitor of system x_c_^−^, leads to GSH depletion and starves GPX4 of its reducing capacity [72,73]. Different from other RCDs with a suicide mission (apoptosis, necroptosis, and pyroptosis), ferroptosis was dubbed a sabotage process where cells die due to disruption of their normal essential processes, such as production of lipid peroxides or aberrant inactivation of the intracellular thiol antioxidant system x_c_^−^/GSH/GPX4 axis [74]. Therefore, experimental ferroptosis models commonly adopt direct GPX4 inhibition via RSL-3 or indirect inhibition through blocking system x_c_^−^ via erastin [75,76,77,78]. The provocateurs of ferroptosis, lipoxygenases (LOX), in particular 15-LOX, predominantly catalyze the enzymatic peroxidation of esterified PUFAs [79,80]. Reactive electrophiles generated from decomposed oxidized PUFA-phospholipids may react with cellular macromolecules and damage both the structural and functional proteins of cell components. Lipid peroxidation of the cellular membrane structure also causes membrane damage and pore formation, limits membrane fluidity, and leads to cell death [44]. Ferrostatin-1 and liproxstatin-1 are potent inhibitors of ferroptosis by their 15-LOX-inhibiting capability combined with radical-trapping antioxidative activity [81]. The distinction of ferroptosis from other RCDs is the absence of cytoplasmic swelling in necrosis [82], the lack of nuclear condensation and chromatin margination in apoptosis [83], and no formation of the double-membrane-enclosed vesicles seen in autophagy [30]. However, characteristic ferroptosis morphological features are mitochondrial presentations, including fragmented mitochondria, mitochondria accumulation around the nucleus, condensed mitochondrial membrane densities, diminished or vanished mitochondria crista, and the rupture of the mitochondrial outer membrane [30]. Different organelles are suggested to play a part in initiation of ferroptosis, including the mitochondria, endoplasmic reticulum, and lysosomes [30]. Thus, cellular iron metabolism, lipid peroxidation, and intracellular antioxidative systems involved in lipid peroxide detoxification, especially the system x_c_^−^/GSH/GPX4 antioxidative axis, are critical regulators of ferroptosis (Figure 1) [30].

### 3.1. Cellular Iron Metabolism

Iron is a paradoxical beneficial and hazardous existence within the cell. Vital for many physiological functions, iron is an essential catalytic center of crucial enzymes, including the ribonucleotide reductase and DNA helicase in DNA replication, the nitric oxide synthases for second messenger transduction, and the cytochrome oxidases in mitochondrial electron transport for ATP production [29,84]. However, the high catalytic activity of iron poses a threat to cell survival in poorly controlled iron-related redox-cycling reactions, as established in ferroptosis [64]. Given the high and often detrimental redox activity of free iron in intracellular and extracellular regions, the transportation and delivery of this high-risk catalyst to its final destinations are under constant control by specialized proteins. The extracellular iron within the plasma is almost exclusively bound to circulating transferrin in the ferric form (Fe^3+^) [85]. The intracellular iron of mammalian cells is stored in two major iron storage pools, the cytoplasm and the mitochondria. Within the mitochondria, Fe^3+^ is stored in mitochondrial ferritin and used in critical anabolic pathways, including heme synthesis and iron–sulfur cluster biogenesis [86]. Circulating Fe^3+^ in the bloodstream is mostly bound to transferrin with one or two atoms of Fe^3+^ per transferrin. Transferrin receptors on cell surfaces bind these iron-bound transferrins, and the transferrin receptor–transferrin-Fe^3+^ complex undergoes endocytosis through clathrin pit formation and is deposited in the endosome. In the acidic environment of the endosome, Fe^3+^ is released from the transferrin and then reduced to ferrous ion (Fe^2+^) by ferric reductases of the six-transmembrane epithelial antigens of the prostate (STEAP) family. Fe^2+^ is then released through the endosome membrane to the cytoplasm through the ferrous iron transmembrane transporter, including divalent metal transporter 1 (DMT1) and the multispecific metal transporter, ZRT/IRT-like protein (ZIP) 8/14 [87]. When systemic iron in the bloodstream is low, extracellular non-transferrin-bound ferric iron can also be converted to Fe^2+^ by cell membrane STEAPs and then directly imported into cells by cell membrane DMT1 or ZIP8/14 [88]. After uptake into the cell and reduction, the free Fe^2+^ in the cytoplasm is collectively referred to as the labile iron pool, and this pool of iron is strictly regulated according to the needs of the cell to be used, stored, or exported to prevent iron overload [89]. The majority of the newly imported cytoplasmic iron (around 70–80%) is stored in the iron storage protein complex, ferritin. The stored iron is then maintained in its non-toxic Fe^3+^ state with the ferroxidase activity of ferritin heavy chains. The minority of the cytoplasmic free Fe^2+^ is used by downstream metabolic pathways, such as being incorporated into cytoplasmic iron-requiring proteins or imported into mitochondria [90]. Due to its high catalytic ability, intracellular Fe^2+^ is coordinated by small molecules and macromolecules to limit its redox activity, and Fe^2+^ is escorted to different intracellular sites to interact with appropriate targets. The primary small molecule proposed to coordinate Fe^2+^ is the reduced GSH, which binds to iron through a thiol ligand contributed by its reduced cysteine residue [91]. GSH is abundant in the cytoplasm, and the complexes formed by GSH and Fe^2+^ are proposed to prevent iron toxicity. With its relatively low iron-binding affinity, GSH is able to release iron at the target site more readily. Iron can also be guided by macromolecular ligands, such as the cytosolic iron chaperones, Poly rC Binding-Protein (PCBP) family, especially PCBP1 and PCBP2 [92]. Working as an adaptor, PCBP2 capture and bring Fe^2+^ into the cytoplasm, when Fe^2+^ is released from channels and enzymes, such as the iron channel DMT1 and heme oxygenase [93]. The Fe^2+^-binding PCBP2 transfers Fe^2+^ to PCBP1 for delivery to client proteins, such as ferritin and non-heme iron enzymes, including 2-oxoglutarate-dependent dioxygenases and monooxygenases of the fatty acid hydroxylase/desaturase type [94,95]. In iron deficiency, iron stored in ferritin is recycled by autophagic degradation of ferritin, called ferritinophagy [96]. To facilitate the process, the autophagic cargo receptor, nuclear receptor coactivator 4 (NCOA4), binds to ferritin and latches to autophagy protein on the autophagosome membrane [97]. Released lysosomal Fe^2+^ is directed to the cytoplasm or into the mitochondria. In surplus, intracellular Fe^2+^ is oxidized to Fe^3+^ and exported by ferroportin, the only known protein that exports intracellular iron in mammals [98]. A balance of iron is essential since excess cytoplasmic/mitochondrial free Fe^2+^ can directly catalyze free radical formation via the Fenton reaction and induce further oxidative damage, including peroxidation of phospholipids in membrane structures. Recent evidence has shown that multiple genes are manipulated in iron metabolism, and among them transferrin, nitrogen fixation 1 (NFS1), iron response element-binding protein 2 (IREB2), and NCOA4 could regulate the ferroptotic process [29]. Additionally, the translational silencing of 15-LOX mRNA is mediated by the specific binding of two PCBPs, and this connects these iron adaptors to ferroptosis [99]. Although the exact role of iron in ferroptosis remains largely unknown, studies have shown that the imbalance between cellular iron import, storage, and export, in favor of iron overload, is pivotal for the induction of ferroptosis and that a sufficient amount of intracellular free Fe^2+^ is necessary in mechanisms leading to the formation of lipid peroxides.

### 3.2. Lipid Peroxidation and Ferroptosis

Fatty acids are essential constituents for membrane structures, and long-chain fatty acids that contain more than two double bonds are called PUFAs, which are mainly obtained from the diet [100]. PUFAs are an essential component of the phospholipid bilayer in biological membranes and influence the dynamics of lipids, protein−lipid interaction, and membrane transport properties [101]. In recent years, a considerable body of evidence has emerged, showing PUFAs as precursors for many signaling lipids and implicating PUFAs in various physiological functions, including inflammation, synaptic plasticity, and age-related neurodegenerative processes [102]. Lipid peroxidation is a process when oxidants, such as free radicals, “steal” electrons from the carbon–carbon double bond in unsaturated lipids and primarily form lipid peroxyl radicals and hydroperoxides [103]. Due to the higher number of double bonds in their structures, PUFAs are especially targeted for ROS attacks and are prone to chain oxidation [103]. Ferroptosis can be further induced by accumulation of oxidized PUFAs aroused from the iron-dependent Fenton reaction upon internal or environmental oxidative stresses [29].

**Figure 2:**
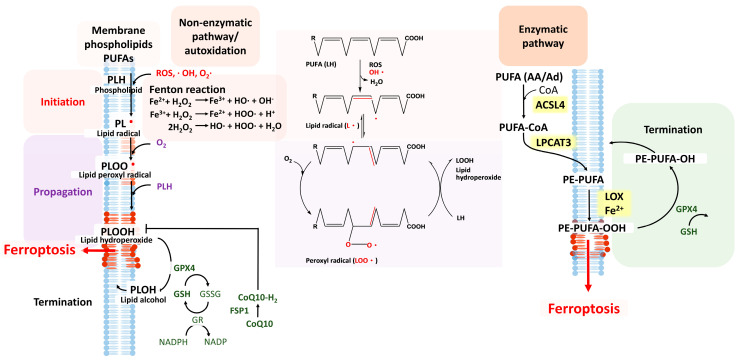
The mechanisms of lipid peroxidation and chemical formula. Lipid peroxidation involves two pathways, the non-enzymatic pathway and the enzymatic pathway. The non-enzymatic pathway includes three steps: initiation, propagation, and termination. In the initiation step, membrane phospholipids are targeted by radicals, such as the hydroxyl radicals, from the Fenton reaction and form lipid radicals. In the propagation step, O_2_ added to the lipid radical forms the lipid peroxyl radical and the addition of phospholipid generates lipid hydroperoxide. This forms a loop, creating new lipid peroxyl radicals, and eventually can lead to ferroptosis. In the termination step, antioxidants, such as the system x_c_^−^/GSH/GPX4 axis and the CoQ10/FSP1 pathway exert, antioxidative effects. GPX4 can directly convert lipid hydroperoxide to lipid alcohol. In the enzymatic pathway, the PUFA is ligated to CoA under ACSL4. LPCAT3 catalyzes PUFA–CoA esterification into PE–PUFA, and the PUFA forms a side chain of the phospholipid. Under the enzyme LOX and the presence of Fe^2+^, PE–PUFA forms toxic PE–PUFA–OOH, which leads to ferroptosis. Antioxidants work in the same way during termination.

Accumulation of lipid peroxides occurs through two pathways, the non-enzymatic and the enzymatic pathway. The non-enzymatic process is an iron-catalyzed spontaneous peroxyl-radical-mediated chain reaction called autoxidation, while the enzymatic pathway is catalyzed by (non-heme) iron-dependent LOXs (Figure 2) [81]. The intracellular hydrogen peroxide (H_2_O_2_) is converted to hydroxyl radicals (HO∙) under the presence of Fe^2+^ via the well-known Fenton reaction [29,104]. In reaction with HO∙ formed in situ, PUFA lipids further undergo spontaneous peroxidation. The process of lipid peroxidation contains three phases, initiation, propagation, and termination, with lipid peroxyl radicals (LOO•) as hazardous by-products. Similar to the generation of HO∙ mentioned before, lipid radicals (L•) can be generated in the presence of Fe^2+^. ROS, reactive nitrogen species, or reactive lipid species extract a hydrogen atom from an allylic carbon, particularly from the membrane PUFAs, to form a L• at the initiation phase [103]. During the propagation phase, an oxygen molecule reacts with L• to form a LOO•, which further extracts another hydrogen atom from allylic carbon to form a new L• and lipid hydroperoxide (LOOH), thus forming a loop to create a new LOO• [27]. To prevent further lipid oxidation, the LOO• can be neutralized by receiving a proton from a proton donor (RH), such as an endogenous antioxidant, to terminate further lipid oxidation (LOO• + RH -> LOOH + R•), the so-called termination phase of lipid peroxidation. This generates the less reactive LOOH and another radical (R•), which can be further degraded. As lipid peroxides accumulate, they can degrade into hydroxy fatty acids or undergo peroxycyclization, during which lipid peroxides decay into reactive toxic aldehydes, including malondialdehyde (MDA) and 4-hydroxynonenal (HNE) [28,105]. MDA and HNE are the most frequently measured biomarkers for lipid peroxidation and oxidative stress [105]. Once formed, MDA and HNE can be reduced to alcohols by aldo-keto reductases or alcohol dehydrogenases or can be oxidized to acids by aldehyde dehydrogenases [105]. Both aldehydes are still highly reactive and can affect gene expression, playing a cytotoxic role [103]. Another means for termination of lipid peroxidation occurs when too much LOO• accumulates and the chance of two peroxyl radicals colliding increases. The collision of two LOO• forms a stable peroxide bridged dimmer and an oxygen molecule (LOO• + LOO• -> LOOL + O_2_). 

In the enzymatic pathway, cellular membrane phospholipids, such as PUFAs, are oxidized through a series of reactions. The Fenton reaction occurs under the presence of toxic Fe^2+^, which activates LOXs, particularly 15-LOX, and catalyzes lipid peroxidation of cell membrane structures, causing membrane destabilization [27]. LOXs are dioxygenases that catalyze the formation of corresponding hydroperoxides from PUFAs. There are six identified LOX isoforms in humans (15-LOX-1, 15-LOX-2, 12-LOX-1, 12-LOX-2, E3-LOX, and 5-LOX), containing nonheme iron via stereospecific peroxidation, and 15-LOX is the main LOX activated during ferroptosis [106]. The substrates of 15-LOX, arachidonoyl (AA)-phosphatidylethanolamine (AA-PE) and adrenoyl (AdA)-PE (AdA-PE), were identified as the main PUFAs undergoing lipid peroxidation in ferroptosis. Upon induction, AA and AdA are ligated to CoA under the catalysis of the required acyl-CoA synthetase long-chain family member 4 (ACSL4) to form AA-CoA and AdA-CoA intermediates. These CoA derivatives are further esterified into phosphatidylethanolamines (AA-PE and AdA-PE) by lysophosphatidylcholine acyltransferase 3 (LPCAT3) [107]. Subsequently, 15-LOX catalyzes the oxidization of AA-PE and AdA-PE in the presence of Fe^2+^ to generate toxic lipid hydroperoxides (PE–PUFA–OOH). PE–PUFA–OOH can subsequently react with an adjacent PUFA to initiate another lipid radical chain reaction as well as serve as ferroptotic signals. PUFAs of both cellular and mitochondrial membrane phospholipids can be oxidized by 15-LOX catalysis and lead to membrane destruction [108]. Should excessive lipid peroxidation overcome the antioxidative system, mainly GPX4, the propagation of lipid peroxidation is unable to proceed to the termination stage, and ferroptosis may occur. The inhibitors of ferroptosis found in studies including ferrostatin-1 and liproxstatin-1 are 15-LOX inhibitors as well as radical-trapping antioxidants that can prevent the autooxidation in the nonenzymatic destruction of membrane PUFAs likely driven by the Fenton reaction [109]. Therefore, maintaining proper redox homeostasis by antioxidative systems, such as the system x_c_^−^/GSH/GPX4 axis, is essential to keep oxidative lipids in check and avoid further ferroptosis.

### 3.3. System x_c_^−^/GSH/GPX4 Antioxidative Axis and Co-Enzyme Q10

The system x_c_^−^/GSH/GPX4 axis is one of the key systems regulating the cellular redox balance and provides a vital defense system to protect cells from oxidative stress. The major antioxidative enzyme that directly reduces lipid hydroperoxides is GPX4, a selenoprotein belonging to the family of glutathione peroxidases [110]. The monomeric enzyme GPX4 can reduce peroxidized lipids either in free or in complex form, including in phospholipids (lipids) and lipoproteins (proteins). The normal function of GPX4 is reliant on GSH as a cofactor for its ability to cycle between oxidized (GS-SG) and reduced (GSH) forms [111]. The antioxidant GSH is synthetized from glutamate, cysteine, and glycine with the help of two enzymes, glutamate cysteine ligase (GCL) and glutathione synthetase (GSS), in two steps. Structured as a simple tripeptide, GSH reduces oxidized intracellular components with its reduced thiol group on the cysteine [112]. The active site of GPx4 shifts between the oxidized and the reduced state in order to maintain its catalytic function. In contact with a peroxide, the active site of GPx4, selenolate (Se-H), is oxidized to selenic acid (Se-OH). Two reduced GSHs then reduce the selenic acid to regenerate GPx4, producing stable non-toxic lipid alcohols (hydroxy-PEs (HO-PEs)) and oxidized glutathione (GS-SG) as end products [113]. Not surprisingly, with its antioxidant potency as well as its sidekick role to GPX4, manipulating GSH production is the widely used experimental procedure for studies on ferroptosis control. For example, activation of GPX4 was found to suppress ferroptosis in a transgenic ALS mouse model [114]. The biosynthesis of GSH is under the control of the transcription factor Nrf2, the key regulator of the cellular response against “stress” [80].

Cysteine is the major functional component of GSH and also the limiting amino acid during GSH synthesis [115]. The intracellular concentration of GSH is fine-tuned via system x_c_^−^, an ATP-dependent glutamate-cystine antiporter on the plasma membrane, which gates cystine import. System x_c_^−^ is composed of the light-chain subunit SLC7A11 and the heavy-chain subunit SLC3A2 linked by a covalent disulfide bond [116]. Highly specific for cystine and glutamate, SLC7A11 is a 12-pass transmembrane protein responsible for the primary transport activity for these amino acids [117], while the single transmembrane protein SLC3A2 primarily functions as a chaperone essential for regulating trafficking of SLC7A11 to the plasma membrane and has been suggested to be required to maintain SLC7A11 stability [118]. After import into the cell, cystine is reduced to cysteine by GSH or thioredoxin reductase 1. In all, inhibiting the function of GPX4 leads to ferroptotic cell death as in the primary discovery of the ferroptotic cell death induced by the system x_c_^−^ inhibitor erastin back in 2003 [28,64]. 

CoQ10, also known as ubiquinone, is an important electron carrier in the mitochondrial ETC and also acts as a lipophilic free-radical-scavenging antioxidant in the cytosol and intracellular membranes [119]. This lipid co-enzyme has a redox-active benzoquinone ring, where Q refers to its quinone chemical group and 10 refers to the number of isoprenyl chemical subunits in the tail required for its specific placement in a biological membrane. Mainly found in the mitochondrial inner membrane, CoQ10 undergoes a redox cycle and shuttles electrons from complexes I and II to complex III of the ETC. Possessing iron–sulfur clusters that only accept one electron at a time, CoQ10 switches between three oxidized states: fully oxidized (ubiquinone), semiquinone (ubisemiquinone), and fully reduced (ubiquinol) [120]. A non-mitochondrial pool of CoQ10 has also been discovered in intracellular membranes, including the plasma membrane, ER, and Golgi apparatus, where CoQ10 plays the role of a lipophilic radical-trapping antioxidant guard [121]. Essential for the antioxidative ability of CoQ10 in stopping the propagation of lipid peroxides, CoQ10 requires assistance from other oxidoreductases to resume its reduced state. One such oxidoreductase, ferroptosis suppressor protein 1 (FSP1), was recently reported by Doll et al. FSP1 is able to complement the ferroptosis induced by the genetic deletion of GPX4 [122]. There are both an N-myristoylation signal and a flavoprotein oxidoreductase domain in FSP1, which are mandatory for its function in suppressing ferroptosis. Bersuker et al. demonstrated that FSP1 localizes to the cell membrane after myristoylation, where it catalyzes the CoQ10 reduction to ubiquinol by NADPH, restoring the antioxidative effects of CoQ10 [123]. Acting as an independent parallel pathway to the system x_c_^−^/GSH/GPX4 axis, this FSP1/CoQ10 pathway prevents irreversible ferroptotic cell death through reducing lipid peroxides.

## 4. Ferroptosis and the Brain

The brain is enriched in long-chain PUFAs, especially in excitable membranes, and lipid peroxidation in the CNS is an important contributor of oxidative stress and leads to cell death via ferroptosis [124]. The blood–brain barrier (BBB) is a selective monolayer of endothelial cells that shield the central nervous system (CNS) from harmful substances in the circulating blood. BBB dysfunction is an early pathophysiological change observed in many neurodegenerative diseases, and studies have shown that lipid peroxidation and iron accumulation are connected to the barrier dysfunction [125,126]. In addition to oxidative damage, neuroinflammation has long been considered to be critically involved in the process of neurodegeneration. Lipid-peroxidation-related oxidative damage can lead to the induction of neuroinflammation. Non-neuronal cells, glia, including the microglia, astrocytes, and oligodendrocytes, are major mediators of neuroinflammation. Glia cells regulate CNS tissue recovery, and their dysfunction is possibly contributory to neurodegeneration [127]. Recent evidence suggests that the brain’s major resident immune cells, microglia, provide disease-modifying regulation of other major glial populations, including astrocytes and oligodendrocytes. Upon activation, microglial cells trigger an inflammatory response, which is maintained and often amplified by astrocytes [128]. This, in turn, exposes neurons to inflammatory mediators that can cause neuronal cell death [129]. The integrated network of neuron–glia communication underlies the ferroptotic perturbations in dopaminergic neurons and forms a vicious circle in promoting PD pathogenesis [66]. Here, we will elaborate on the involvement of ferroptosis at the blood–brain barrier and glia cells.

### 4.1. Ferroptosis and the Blood–Brain Barrier

The blood–brain barrier (BBB) refers to the highly selective semipermeable border that surrounds most of the blood vessels in the CNS. It acts as a shield between the bloodstream and the extracellular space of the brain and precisely controls substances to enter and leave the nervous tissue [130]. The BBB is composed of several cell types, including the monolayer of endothelial cells that form the wall of the blood vessels, the CNS mural cells called pericytes that partially cover the outside of the endothelial cells, and the glial cells named astrocytes whose processes, end-feet, sheath the blood vessels [131]. A central component in the structure of the BBB is the tight junction connecting the endothelial cells, limiting paracellular permeability [132]. Damage to the BBB can cause dysfunction of the tight junctions, transport proteins, and leukocyte adhesion molecules, further causing brain edema, perturbed ion homeostasis, altered signaling, and immune infiltration, and finally can lead to neuronal death. The evidence of ferroptosis occurring at the BBB was found as studies have demonstrated concurrent increased iron levels, increased lipid peroxidation, and decreased GSH and CoQ10 levels at the BBB. Iron is an important cofactor in multiple neuronal activities. Under normal physiological conditions, iron levels in the brain are tightly regulated by strict restriction of iron transport across the BBB and the feedback loops of iron levels in the CNS [37]. A recent study demonstrated in an established BBB injury model of organophosphate paraoxon on a stem-cell-derived in vitro human BBB system that organophosphate-induced BBB breakdown and oxidative stress could be rescued by the iron chelator desferal (DFO). The group reported that the increase in brain endothelial-cell-free Fe^2+^ in the LIP was responsible for BBB damage and that DFO abrogated this effect by chelating the free Fe^2+^ in the LIP, inducing the expression of hypoxia-induced factor 2α (HIF2α) and reversing the loss of Ve-cadherin expression [133]. This suggests that ferroptosis plays a crucial role in damaging the BBB and therefore could be hindered by iron-chelating agents. The PUFA AA is a major constituent of brain lipids and was also found to cause BBB damage by increasing ROS through LOX or cyclooxygenase (COX) signaling pathways [134,135,136]. AA is a major source of prostaglandin E2 (PGE2), and PGE2 was reported to be associated with dysruption of the BBB in a rat model [137]. In addition, systemic administration of the PGE2 receptor antagonist was found to reduce brain inflammation, prevent blood–brain barrier opening, and provide neuroprotection in the hippocampus in a mouse status epilepticus model [138]. AA-mediated permeability in a confluent monolayers of human brain microvascular endothelial cells could be attenuated by specific COX2 inhibitors and EP3 and EP4 receptor antagonists [135]. In a traumatic brain injury mouse model, the lipophilic antioxidant ferrostatin-1 could significantly reduce brain microvascular endothelial cells death, decrease BBB permeability, and alleviate tight-junction loss [139]. These studies implicate ferroptosis factors, including iron, lipids, and ROS, in the breakdown of the BBB. With the genome-wide CRISPR–Cas9-mediated suppressor screens, cytochrome p450 (CYP) enzymes were found to be involved in lipid-peroxidation-caused iron-dependent ferroptosis [140], and some of these enzymes were located at the BBB interface [141]. The phospholipid transfer protein (PLTP) regulates lipid metabolism and is highly expressed in the BBB. In PLTP-deficient mice, BBB permeability increases with decreased expression of tight-junction proteins occludin, zona occludens-1 (ZO-1), and claudin-5 in brain vessels. A concurrent increase in ROS and the lipid peroxidation marker HNE and reduced antioxidative SOD activity was noted [142]. Within the CNS, the synthesis of GSH is limited by the availability of the sulfhydryl amino acid L-cysteine, which primarily crosses the BBB through the BBB transporters. Transporters that can transport GSH and GSSG include multidrug resistance proteins (MRPs/Mrps) [143]. Other means for acquiring GSH in BBB endotherlial cells has recently been reported through transport from the non-neuronal glia, astrocytes, which contain comparatively high levels of GSH [144]. Under time-of-flight mass spectrometry (TOF-MS), astrocytes were found to constantly shuttle GSH to endothelial cells under resting conditions, and this flux was accelerated by injury in rat brain cells [144]. Thus, the progression of BBB injury can accelerate the progress of neurodegenerative disease, and the prevention of BBB damage can be prevented by increased endothelial GSH [145].

### 4.2. Potential Cross Talk between Neurons and Glia Cells involving Ferroptosis 

As stated before, both glial cell activation and iron dyshomeostasis are major contributors to PD pathogenesis. They can reciprocally influence each other and boost dopaminergic neuron degeneration. Glia activation can further promote iron dyshomeostasis and aggravate microglial activation. Ferroptosis may thus take part in the interaction between glia and neurons and thus modulate the pathogenetic process of PD, as reviewed by Wang [66]. Three main types of glia cells (microglia, astrocytes, and oligodendrocytes) act via different pathways to attenuate oxidative stress in neuronal networks and prevent excessive amounts of ROS from entering neurons [146]. Briefly, activated microglia and astrocytes promote iron accumulation in neurons by upregulation of DMT1 and downregulation of ferroportin through the release of inflammatory cytokines [147,148]. Astrocytes can also release hepcidin and prevent iron release from neurons through ferroportin under the stimulation from the inflammatory cytokine IL-6 [149]. Activated astrocytes secrete BDNF and GDNF and downregulate DMT1, which reduces iron accumulation in neurons [150]. Upregulation of the transcription factor Nrf2 in astrocytes also contributes to neuronal resistance to oxidative stress [151]. Oligodendrocytes secrete ferritin heavy chains as an antioxidant defense mechanism for neurons against iron-induced cytotoxicity. Both astrocytes and oligodendrocytes can effectively control the level of glutamate in the synaptic cleft by regulating GSH synthesis and inhibiting neuronal system x_c_^−^ [152]. Thus, three types of glia regulate neuroinflammatory processes, intercelluar oxidative stress, and this unique type of iron dependent cell death, ferroptosis [66].

## 5. Iron Deposition, α-Synuclein Aggregation, Lipid Peroxidation, and Ferroptosis in PD

To date, a cure has still not been found for the progressive dopaminergic neuronal loss in the SN to stop the degeneration in PD. Progressive neuron loss has been linked to pathological α-synuclein aggregation in Lewy bodies, with oxidative stress playing a prominent role. The characteristic pathological changes observed in the SN of patients with PD include increased iron deposition, increased lipid peroxidation, and defects in the system x_c_^−^/GSH/GPX4 antioxidative axis, which is highly consistent with features of the iron- and lipid-peroxidation-dependent ferroptosis [66]. Thus, preventing neural death due to ferroptosis may hinder the progression of this disease.

### 5.1. Iron Deposition in PD

In the late 1980s, the elevation of iron in the SN of patients with PD compared to controls was reported [153,154,155]. Sofic et al. compared the iron concentration in different brain regions of patients with PD and found that in contrast to elevated iron in the SN, no significant difference in the levels of Fe^3+^ and total iron was noted in other areas of the brain, including Brodmann area 21, the hippocampus, the putamen, and the globus pallidus [156,157]. Additionally, post-mortem studies on patients with PD have also demonstrated sequestration of iron in Lewy bodies in the neurons of the SN pars compacta [158,159]. These increased iron levels in the SNpc of brains of patients with PD have since been demonstrated by a variety of modalities, including inductively coupled plasma spectroscopy [155], magnetic resonance imaging [160], laser microprobe mass analysis [161], susceptibility-weighted imaging [162,163], and enhanced T2-star-weighted angiography [32,164]. In correlation to the progressive nature of PD, Biondetti et al. demonstrated a spatiotemporal relationship between dopaminergic striatal dysfunction and increased nigral iron content via neuromelanin and iron-sensitive MRI and dopamine transporter single-photon emission tomography in a 2-year cohort study ICEBERG [165]. These studies accentuate the role of iron accumulation in dopaminergic degeneration at the SN during PD progression. 

### 5.2. α-Synuclein Aggregation and Iron Deposition

α-synuclein can be conformed into the toxic species of oligomers or fibrils under environmental perturbations, such as oxidative stress, [166], and the kinetics for α-synuclein fibrillation was noted to be further enhanced under the presence of metal ions, including Fe^2+^ [167]. Iron deposition in the midbrain was found to be colocalized with α-synuclein, which supports that a higher concentration of iron in the substatia nigra may contribute to the formation of α-synuclein aggregation and Lewy body pathology [168]. α-synuclein, the main component of Lewy bodies, contains 140 amino acids with an N-terminal region that can adopt α-helical folding, an amyloid-like fibril-prone central domain, and a C-terminal disordered region acting like a shield to prevent aggregation of the central domain [169]. Vulnerability to iron accumulation in the SN was demonstrated in the overexpression of mutant human A53T α-synuclein in transgenic PD mice, and the iron deposition correlates with α-synuclein aggregation and the development of age-dependent motor deficits [170]. In a drosophila model with parkinsonian phenotypes (over-expressing A53T, A30P, and wild-type (WT) α-synuclein), Wu et al. demonstrated that iron induces more severe motor deficits and selective neuron loss in the dopaminergic PPM3 cluster in the brains of mutant α-synuclein-expressing flies [171]. The triplication of the α-synuclein-encoding gene SNCA was found to incorporate excess α-synuclein oligomers into membranes, induce lipid peroxidation, and drive ferroptosis, while the addition of the antioxidant linolenic acid (D4-Lnn) or vitamin E inhibits lipid peroxidation and prevents death in human-induced pluripotent stem cell (iPSC)-derived neurons [31]. The importance of iron in enhancing the spreading of α-synuclein between dopaminergic neuron death was shown by Xiao et al., who demonstrated that the addition of Fe^2+^ inhibits autophagosome–lysosome fusion and increases α-synuclein spreading between these cells [172]. Interestingly, different routes of iron chelator application can also provide neuroprotection. Intranasal administration of the iron chelator deferoxamine decreased the number of the pathological α-synuclein formations and showed an overall partial improvement in motor behavior in a rat PD model study [173]. Therefore, iron and α-synuclein seem to form a vicious cycle, and strategies targeting lowering iron deposition may be a potent attenuator for α-synuclein accumulation and spreading.

### 5.3. Lipid Peroxidation, Ferroptosis, and PD

Oxidative stress has long been linked to the progressive dopaminergic neuronal loss in PD. Lipid peroxidation, the main player in ferroptosis, has been demonstrated in cellular and animal PD models using various mitochondrial complex I inhibitors, including MPTP, rotenone, plaquenil, and 6-OHDA [174]. PUFA levels were found to decrease in the SN, along with increased MDA levels, suggesting increased lipid peroxidation and oxidative damage in the dopaminergic neurons of patients with PD [175]. Additionally, Fedorova et al. demonstrated a reciprocal increase in lipid peroxidation products with clinical PD-advancing stages in the peripheral blood of 240 patients with PD [33]. A reduction in antioxidative protective systems was also found in a post-mortem study, and the SN from patients with PD possessed reduced GSH levels compared to the control group [176,177]. Involvement of the antioxidative protective mechanism system x_c_^−^ was also documented in a recent study by Vallerga et al. From this blood-based methylome-wide association study of PD involving meta-analysis of 229 K CpG probes in 1132 cases and 999 controls from two independent cohorts, the group identified that the cg06690548 on chromosome 4 is significantly associated with PD and cg06690548 hypermethylation in PD is associated with downregulation of the SLC7A11 gene, which codes for system x_c_^−^ [178]. These reports show that increased ROS in the brain with enhanced lipid peroxidation and depletion of GSH stores may be a factor that spurs the progression of PD. The autosomal recessive PD risk gene DJ-1 has also been reported to be implicated in causing ferroptotic cell death. As reported by Jiang, the over-expression of DJ-1 significantly inhibits erastin-triggered ferroptotic cell death, and this suppression of ferroptosis can be eliminated when the C-terminus of DJ-1 is mutated [179]. The protein encoded by DJ-1 possesses antioxidative effects via directly eliminating ROS through oxidizing itself at the Cys106 residue to –SO_2_ or –SO_3_ [180]. This protection by DJ-1 was suggested to be through maintaining the generation of cysteine, even under the blockage of system x_c_^−^-mediated cysteine import, ensuring the biosynthesis of GSH. The suppression of DJ-1 markedly increased the sensitivity of cells to ferroptosis induction [181]. Anti-ferroptotic agents also protect dopaminergic neurons from ferropstosis in PD models; for example, ferrostatin-1 or deferiprone was demonstrated to be able to hinder cell death, restore tyrosine hydroxylase expression, and alleviate locomotor behavioral deficits [182]. The novel orally bioavailable iron chelator PBT434 (8-hydroxyquinazolin-4(3H)-one) that could sufficiently bind iron without disrupting physiological iron homeostasis could prevent loss of neurons in the SN pars compacta, lower nigral α-synuclein accumulation, and rescue motor performance in experimental animals exposed to 6-OHDA or MPTP. These improvements were associated with decreased oxidative damage and upregulated ferroportin and DJ-1 expression, as documented by Finkelstein et al. [183]. Hence, the development of anti-ferroptotic agents may provide opportunities to facilitate protective therapy for PD.

## 6. Clinical Trials of Anti-Ferroptotic Agents in PD Treatment

Clinical trials using medications that may possess potential anti-ferroptotic effects on patients with PD are still at an early stage, and promising results are dawning. In two phase II randomized, double-blind placebo-controlled clinical trials (NCT00943748, NCT01539837), the high-affinity iron chelator deferoxamine was demonstrated to decrease SN iron accumulation and reduce the progression of motor symptoms in patients with PD, although with side effects noting decreased white blood cell counts, gastrointestinal upset, and joint pain [184,185,186]. A third trial, the sky study, evaluated the effect of four different dosages of deferiprone plus a placebo group on 140 patients with early-stage PD currently on antiparkinsonian medication, and though completed, results are yet to be published (NCT02728843). Due to the primitive success of these studies, the FAIR PARK II study, a more extensive European multicenter parallel-group placebo RCT with 372 patients with untreated de novo PD enrolled, was conducted to test the protective effect of deferiprone on patients with PD (NCT02655315, doi 10.3030/633190). The iron content in the nigrostriatal pathway significantly reduced under deferiprone, the volume in the bilateral putamen and caudate nucleus reduced in the placebo group, and there was no significant change in the dopamine transporter (DaT) density between the placebo and deferiprone-treated groups. Surprisingly, preliminary results of this study demonstrated that the mean change in the total MDS-UPDRS score was 16.7 points with deferiprone and 6.3 points with the placebo after 36-week treatment and another 4-week post-treatment monitoring. The unexpected result of deferiprone to worsen the handicap of persons without dopaminergic treatment was speculated to be due to iron being critical for dopamine synthesis (tyrosine hydroxylase cofactor) and mitochondrial oxidative phosphorylation. The authors proposed that iron accumulation is a powerful short-term compensatory mechanism for increasing dopamine synthesis but possibly at the expense of long-term worsening iron-related cell death. Authors also suggest another clinical trial with a longer duration of treatment to prove the concept that the use of iron chelators in combination with dopaminergic therapy could avoid the long-term compensatory iron accumulation caused by dopamine synthesis (NCT02655315, doi 10.3030/633190). These clinical trials of iron chelators on patients with PD show inconsistent results, and thus, further trials on iron chelators as an add-on to dopaminergic regimens in patients with PD are required. Apart from iron chelators, preliminary evidence suggested that Cu(II)ATSM, a Cu^II^ complex with radical-trapping activity, can exert protection in PD by preventing lipid peroxidation in patients with early idiopathic PD in a phase I study (NCT03204929) [66].

In addition, antioxidative therapies targeting ferroptotic pathophysiology, including GSH, N-acetylcysteine (NAC), and CoQ10, have also been shown to be beneficial to PD. A summary of these clinical trials is shown in Table 2. A phase IIb study investigated the effect of intranasal GSH in 45 patients with PD and demonstrated improvement in motor function over baseline (NCT02424708) [187]. In addition, other small clinical trials testing GSH and the GSH precursor NAC in PD have also demonstrated symptomatic improvement [187,188,189]. However, there are some other clinical trials on GSH use in patients with PD that have shown inconclusive or negative results (Table 2). More recently, a new RCT initiated in 2022 is recruiting participants to investigate the effect of intranasal GSH and insulin as an add-on therapy for PD (NCT05266417). Apart from GSH, the antioxidant CoQ10 has also received much attention in the hope of delaying PD progression. Early in 2002, a multicenter RCT reported the benefit of CoQ10 in slowing motor symptom progression in PD, with a positive protective trend in the three groups with CoQ10 dosages of 300, 600, and 1200 mg/d (NCT00004731) [190]. However, in subsequent clinical trials with larger PD cohorts, CoQ10 did not reach the primary endpoints for functional scales (Table 2) [190,191,192,193,194]. Larger phase III studies of the antioxidants CoQ10, GSH, and NAC may be warranted in hopes of evidence for disease modification in PD.

## 7. Conclusions

Innovative research has provided advanced insight into our understanding of ferroptosis, incorporating the iron metabolism, lipid peroxidation, and antioxidant defense system x_c_^−^/GSH/GPX4 axis. Overlapping with ferroptosis is the pathological characteristics of PD, which include the association between neuronal death, accumulation of iron, and α-synuclein aggregation in the SN. The PD-susceptible gene *DJ-1* and *SNCA* mutation are found to trigger ferroptosis. In addition, treatment targeting anti-ferroptotic mechanisms has demonstrated motor improvements in a PD animal model. Over the past decade, clinical trials on ferroptosis-counteracting agents in PD, especially emphasizing iron chelators and anti-oxidative agents, such as GSH, NAC, and CoQ10, are still ongoing. Although the efficacy of these ferroptosis inhibitors in PD is still unsettled, future exploration on the use of these anti-ferroptotic agents in decelerating PD progression is mandatory and deserves further investigation.

## Figures and Tables

**Table 1:** Cell death modalities, their morphological features, and key participants.

Cell Death Pathway	Morphology		Mechanisms and Key Participating Molecules		Ref.
**Apoptosis** **I. Extrinsic** **II. Intrinsic** **(mitochondrial)**	Cytoplasmic shrinkage		**Extrinsic: death receptor** **pathway**	**Common executioner:** Caspase-3/7 PARP cleavage	[34,35,36,37]
	Chromatin condensation (pyknosis)		Death receptors		
	Nuclear fragmentation (karyorrhexis)		Caspase-8		
	Plasma membrane blebbing		**Intrinsic**: mitochondrial pathway		
			Bcl-2, Bax, Bak,		
	Formation of small vesicles (apoptotic bodies) that are efficiently taken up by neighboring cells with phagocytic activity and degraded within lysosomes		MOMP, cytochrome c, Smac, AIF, APAF-1		
			Caspase-9		
**Autophagy**	Double-membrane-enclosed vesicles		ATGs, Bcl-2, ULK1, PI3KIII, P62, LC3, lysosome		[38]
	Extensive cytoplasmic vacuolization				
	Phagocytic uptake				
	Lysosomal degradation				
**Necrosis**	Cytoplasmic and organelle swelling		Acute cell damage, such as trauma, or severe hypoxia		[39]
	Rupturing of plasma membrane				
	Necrotic-like	Apoptotic-like			
**Necroptosis** **(regulated** **necrosis)**	Cytoplasmic swelling		RIPK3, MLKL (alternative cell death when caspase-8 is blocked)		[39,40,41]
	Rupturing of plasma membrane				
**Pyroptosis**	Rupturing of plasma membrane	Chromatin condensation	NLRs, AIM2, IL-1β, IL-18, caspase-1,		[42,43]
			caspase-11/4/5,		
		Plasma membrane blebbing	Gasdermin cleavage,		
			mitochondrial ROS		
**Ferroptosis**	Rupturing of plasma membrane		Iron overloading, lipid peroxidation (pro-ferroptotic)		[26,27,44]
	Shrinkage of mitochondria				
	Decreased mitochondrial cristae		System X_C_^−^, GPX4, GSH, CoQ10, FSP1 (anti-ferroptotic)		
	Ruptured mitochondrial membrane				

White/gray: tolerogenic cell death (apoptosis, autophagy); orange: immunogenic (necrosis, necroptosis, pyroptosis, ferroptosis).

**Table 2:** Clinical trials of candidate agents targeting ferroptosis in PD.

Mechanism of Action	Agent Dosing	Trial Phase (Participant Number), (Location), PD Patient Status, Treatment Period, and Outcome	NCT Registration Number, (Year) Reference
Iron chelating agent	**DFP** 30 mg/kg/d, placebo	Phase II, III Pilot, DBRCT (40) (France) early-stage PD, on stabilized dopaminergic regimens 12 months, **Reduced iron levels in the SN, and improved motor function in patient with PD**	FAIR-PARK-I NCT00943748, (2009–2012) [184,185]
	**DFP** 20 mg/kg/d, 30 mg/kg/d, placebo	Phase II DBRCT (22) (London) early-stage PD, on stabilized PD regimens 6 months, **Reduced iron levels in the SN** **DFP-treated patients showed a trend for improvement in motor-UPDRS scores and quality of life, this did not reach significance**	DeferipronPD NCT01539837 (2012–2014) [186]
	**DFP** 20 mg/kg/d, placebo	Phase II DBRCT (372) (European multicenter) untreated de novo patients with PD 36 weeks **Preliminary data:** **Reduced iron levels in the SN** **Change of smaller volume of putamen and caudate in placebo** **No change in dopamine transporter density** **Worsened handicap of DFP group compared with placebo**	FAIR PARK II NCT02655315 (2015–2021) doi 10.3030/633190
	**DFP** 300 mg/d, 600 mg/d, 900 mg/d, 1200 mg/d, placebo	Phase II Dose ranging DBRCT (140) (UK) early-stage PD, on stabilized PD regimens 9 months **Completed, No found publication**	SKY NCT02728843 (2016–2019)
Radical scavenger	**Oral Cu(II)ATSM, a CuII complex** (diacetylbis(N(4)-methylthiosemicarbazonato) copper(II)) (12 mg/day+	Phase I open-label dose escalation clinical trial (31) (Australia) early-stage PD, on stabilized PD regimens one to six 28-day treatment cycles **Completed, No found publication**	NCT03204929 (2017–2019)
Antioxidants	**IV GSH** 1400 mg TIW	Phase II DBRCT (20) (United States) PD, on stabilized PD regimens 4 weeks **Completed, No found publication**	NCT01177319 (2003–2007)
	**Intranasal GSH** 300 mg/d, 600 mg/d, saline intranasal delivery	Phase 1 DBRCT (34) (United States) PD, on stabilized PD regimens 4 weeks **Completed, No found publication**	NCT01398748 (2012–2016)
	**Nasal spray reduced GSH** 100 mg/d, 200 mg/d, placebo	Phase IIb DBRCT (45) (United States) PD, on stabilized PD regimens 12 weeks **Non superior to placebo in motor function**	(in)GSH NCT02424708 (2015–2016) [187]
	**Intranasal INS-GSH** twice daily, Placebo	phase II DBRCT (56) (United States) early-stage PD, on stabilized PD regimens **Recruiting**	NOSE-PD NCT05266417 (2022-est. 2024)
	**NAC** 1800 mg/d, 3600 mg/day, placebo	Phase II DBRCT (50) (United States) PD, on stabilized PD regimens 30 days **Completed, No found publication**	NAC for PD NCT01470027 (2012–2016)
	**NAC** total 6000 mg/d	Phase II open-label clinical trial (8) (United States) PD, on stabilized PD regimens 28 days **Completed, No found publication**	NCT02212678 (2014–2015)
	**CoQ10** 300 mg/d, 600 mg/d, 1200 mg/day, placebo	Phase II DBRCT (80) (multicenter, United States) early-stage PD, no previous use of PD regimen 16 months **Improved motor function, with highest benefit in the highest-dosing group**	NCT00004731 (1998–2002) [190]
	**CoQ10** (add-on) 300 mg/d, placebo	Phase II, III DBRCT (131) (multicenter, Germany) midstage PD, on stabilized PD regimens 3 months **Add-on CoQ10 does not display symptomatic effects in midstage Parkinson disease**	NCT00180037 (2003–2009) [193]
	**CoQ10** 2400 mg/d, GPI-1485 4000 mg/d, placebo	Phase II DBRCT (195) (United States, Canada 42 trial centers) early-stage PD, no use of PD regimen, but maybe be added to pt remaining in the study 12 months **The primary outcome measure did not meet the prespecified criteria. However, authors question the appropriateness of the predetermined definition of futility.**	NCT00076492 NCT00063193 (2004–2007) [194]
	**CoQ10** 1200 mg/d + vitamin E, 2400 mg/d + vitamin E, placebo + vitamin E	Phase III DBRCT (600) (United States, Canada 67 North American sites) early-stage PD, no use of PD regimen 16 months or until a disability requiring dopaminergic treatment **Early terminated due to no evidence of clinical benefit**	QE3 NCT00740714 (2008–2011) [192]
	**CoQ10** 400 mg/d, 800 mg/d, 1200 mg/d, 2400 mg/day	Phase II, III 10-week dose escalation open-label clinical trial (20) (Singapore) early-stage PD, on stabilized PD regimens Each dose 2 weeks **Improvements in the activities of daily living and motor components of the UPDRS**	NCT01892176 (2012) [191]
	**Oral EPI-743** (vitamin E based) 600 mg/d, 1200 mg/d	Phase IIa open-label clinical trial (10-->7) (United States) PD 6 months **EPI-743 improved motor functions**	NCT01923584 (2013–2015) [192]
	**CoQ10 analogue** **idebenone** 30 mg/tab 1# TID, placebo	Phase II, III DBRCT (180) (multicenter, China) early-stage PD, no use of PD regimen For prodromal PD 24 months **Completed, No found publication**	SEASEiPPD NCT04152655 (2020–2022)

Abbreviations: CoQ10, coenzyme Q10; DB, double-blind; DFP, deferiprone; GPI-1485: a neuroimmunophilin-ligand compound; GSH, glutathione; NAC, N-acetylcysteine; RCT, randomized clinical trial.

## Abbreviations

**AA**	Arachidonic acid
**AdA**	Adrenoyl
**ACD**	Accidental cell death
**ACSL4**	Acyl-CoA synthetase long-chain family member 4
**AIF**	Apoptosis-inducing factor
**APAF1**	Apoptotic-peptidase-activating factor 1
**ATG**	Autophagy-related protein
**ATP**	Adenosine triphosphate
**ATP13A2**	ATPase type 13A2
**BAK** **/** **BAX**	Bcl2-antagonist killer/bcl-2-associated x protein
**CNS**	Central nervous system
**CoQ10**	Co-enzyme Q10
**COX**	Cyclooxygenase
**Cu**	Copper
**DAMP**	Damage-associated molecular patterns
**DJ-1**	Daisuke-junko-1
**DMT1**	Divalent metal transporter 1
**ETC**	Electron transport chain
**FBXO7**	F-box only protein 7
**FSP1**	Ferroptosis suppressor protein 1
**Fe^2+^**	Ferrous ion
**Fe^3+^**	Ferric iron
**GPX 4**	Glutathione peroxidase 4
**GSH**	Glutathione
**HNE**	4-hydroxynonenal
**HO•**	Hydroxy radical
**H_2_O_2_**	Hydrogen peroxide
**LC3**	Microtubule-associated protein light chain 3
**LH (PLH)**	Lipid (phospholipid)
**L•**	Lipid radical
**LOH**	Lipid alcohol
**LOO**	Lipid peroxyl radical
**LOOH**	Lipid hydroperoxide
**LOX**	Lipoxygenases
**LPCAT3**	Lysophosphatidylcholine acyltransferase 3
**LRRK2**	Leucine rich repeat kinase 2
**MDA**	Malondialdehyde
**MLKL**	Mixed lineage kinase domain-like protein
**MOMP**	Mitochondrial outer membrane permeabilization
**MPTP**	1-Methyl-4-phenyl-1,2,3,6-tetrahydropyridine
**MRPs**	Multidrug resistance proteins
**NADPH**	Reduced nicotinamide adenine dinucleotide phosphate
**NADP**	Nicotinamide adenine dinucleotide phosphate
**NCCD**	Nomenclature Committee on Cell Death
**NCOA4**	Nuclear receptor coactivator 4
**NLR**	NOD-like receptors
**OXPHOS**	Oxidative phosphorylation
**6-OHDA**	6-Hydroxydopamine
**PARP**	Poly(ADP-ribose)-polymerase
**PCBP**	Poly rC binding-protein
**PCD**	Programmed cell death
**PD**	Parkinson disease
**PE**	Phosphatidylethanolamine
**PI3KIII**	PI3K, class III phosphoinositide 3-kinase
**PINK1**	Phosphatase and tensin homologue (PTEN)-induced putative kinase 1
**PLTP**	Phospholipid transfer protein
**PUFA**	Polyunsaturated fatty acid
**RCD**	Regulated cell death
**RCT**	Randomized clinical trial
**RIPK3**	Receptor-interacting protein kinase 3
**ROS**	Reactive oxygen species
**RSL-3**	Ras-selective lethal small molecule 3
**STEAP**	Six-transmembrane epithelial antigens of the prostate
**SOD**	Superoxide dismutase
**SN**	Substantia nigra
**TNF**	Tumor necrosis factor
**ULK1**	Unc-51 like autophagy activating kinase 1
**ZIP 8/14**	ZRT/IRT-like protein 8/14
